# Psycholinguistic Investigation of the Immediate Interpretation of Plural Nouns in the Scope of Sentential Negation in Polish

**DOI:** 10.1007/s10936-020-09706-7

**Published:** 2020-06-03

**Authors:** Piotr Gulgowski, Joanna Błaszczak

**Affiliations:** Institute of English Studies, University of Wrocław, Wrocław, Poland

**Keywords:** Exclusive/inclusive reading, Negation, Number, Plural, Stroop effect

## Abstract

The number meaning of grammatically plural nouns is to some extent context sensitive. In negative sentences, plural nouns typically receive an inclusive reading referring to any number of individuals (one or many). This contrasts with their more frequent exclusive reading referring to a group of two or more individuals. The present study investigated whether a plural noun in a negative sentence is treated as inclusive immediately when it is encountered or whether this interpretation is delayed. In an experiment using a technique based on a numerical variant of the Stroop effect (Berent et al. in J Mem Lang 53:342–358, 2005. 10.1016/j.jml.2005.05.002; Patson and Warren in J Exp Psychol Learn Mem Cogn 36(3):782–789, 2010. 10.1037/a0018783), participants counted visually presented singular and plural Polish nouns embedded in either affirmative or negative sentences. The nouns were displayed once or as two copies. Plural nouns were easier to count when they were repeated twice on the screen than when only one copy was displayed. For singular nouns this pattern was reversed and the effect was weaker. Crucially, no difference was found for plural nouns appearing in affirmative and negative sentences. This indicated that an inclusive (“one or more”) reading of plural nouns in the scope of sentential negation was not immediate. The results are in line with past research suggesting that the semantic processing of a negative sentence may proceed in two phases (Fischler et al. in Psychophysiology 20(4):400–409, 1983. 10.1111/j.1469-8986.1983.tb00920.x; Kaup et al. in J Pragmat 38:1033–1050, 2006. 10.1016/j.pragma.2005.09.012; Lüdtke et al. in J Cogn Neurosci 20(8):1355–1370, 2008. 10.1162/jocn.2008.20093; Spychalska in Proceedings of the 2011 ESSLLI student session, 2011).

## Introduction

Grammatical number allows language users to express meanings related to the numerosity of objects under discussion using systematic contrasts, like dog vs. dogs in English. However, number markers are not always reliable indicators of number meaning. The plural suffix usually indicates that the noun refers to a group of two or more things. This is known as the exclusive plural reading. Yet in negative sentences plural nouns are typically interpreted as referring to any number of things, one or many. This is known as the inclusive plural reading. The inclusive/exclusive distinction has been studied by both theoretical linguists and psycholinguists. One aspect of the distinction that has attracted relatively little attention is the question whether the inclusive reading of a plural noun in the scope of sentential negation is available already at the point when they are first encountered in the sentence or whether the inclusive reading arises from the interpretation of the sentence as a whole. Investigating this issue is the goal of the present study. Answering this question should lead to a better understanding of both negation and grammatical number processing. It should also reveal more about the timing at which different sources of information become available to comprehenders, which is part of the research on incrementality in language processing.

This paper is structured as follows. First, “Background” provides the background information, including a brief introduction of grammatical number, exclusive and inclusive plural readings, negation and parser incrementality. Previous theoretical and experimental research is discussed. Next, “Numerical Stroop Interference” section presents the experimental technique chosen for the present study and describes the past studies which introduced and developed this method. “Present Study: Research Problem, Hypotheses and Predictions” section discusses the research problem, hypotheses and predictions. “Experiment” section presents the experiment itself, including the materials, procedure, participants and results. Finally, “Discussion” section offers a discussion of the findings.

## Background

### Number as a Grammatical Category

Number is a grammatical category found in many languages. It is inflectional rather than derivational, i.e., it adds extra information to the meaning of a word without altering its core semantic features or changing its syntactic category.^1^ It is a nominal category relevant for the form and interpretation of nouns and pronouns.^2^ Semantically, it is quantity-related, allowing speakers to communicate how many things they have in mind.^3^ This is accomplished either by modifying the form of a word or by introducing a separate number element. In many languages, number enters into morphosyntactic relations between sentence constituents in the form of various types of agreement, as illustrated below.

**Table:** 

(1)	A duck swims/Ducks swim.	[subject-verb]
(2)	this squirrel/these squirrels	[determiner-noun]

**Table:** 

(3)	nudny	artykuł	/	nudne	artykuły	[adjective-noun] [Polish]
	boring.sg	paper	/	boring.pl	papers	

Although linking number forms with number meanings seems fairly straightforward, it turns out to be more problematic. It is true that, in general, singular forms tend to refer to single and plural forms to multiple entities, but sometimes it is not the case. The ultimate number interpretation of a noun is a function of its grammatical number and the context in which it occurs. For example, a singular noun in the scope of the distributive quantifier *each* may refer to a plurality of objects.

**Table:** 

(4)	Each of the men carried a box.

One of the readings of sentence (4) is that there were multiple men carrying multiple boxes (a reading where multiple men took turns carrying a single box is also available, although perhaps less natural).

### Exclusive and Inclusive Plural Reading

The number meaning of grammatically plural nouns is similarly context sensitive. In certain expressions, most notably questions, negative sentences and conditional constructions, plural nouns are typically understood as referring not to a group of two or more individuals (exclusive plural interpretation, dominant in declarative affirmative sentences) but to any number of individuals, one or many, as long as it is not zero (inclusive plural interpretation).

**Table:** 

(5)	a.	Have you seen any squirrels?
		[I can answer “yes” truthfully even if I saw just one squirrel.]
	b.	*I haven’t seen any squirrels.*
		[Sentence is false if I saw even a single squirrel.]
	c.	*If you see any squirrels, let me know.*
		[The speaker wants to be notified if at least one squirrel was seen.]

It has been proposed that an important factor determining the inclusive or exclusive reading of plural nouns is monotonicity (Farkas and de Swart 2010; Sauerland et al. 2005; Zweig 2009). Monotonicity is a logical property related to the direction of inferences associated with a given construction. A predicate which allows inferences from a subset to a superset is called upward monotone, whereas a predicate allowing inferences in the opposite direction is called downward monotone.^4^

**Table:** 

(6)	I have an apple	→	I have a fruit	[upward monotone]
	I have a fruit	↛	I have an apple

**Table:** 

(7)	I don’t have an apple	↛	I don’t have a fruit	[downward monotone]
	I don’t have a fruit	→	I don’t have an apple	

The exclusive interpretation of plural nouns seems to arise in upward monotone contexts. Downward monotone contexts are associated with the inclusive interpretation.

This claim has been tested by Anand et al. (2011) in a series of experiments using the image verification technique. Participants read sentences accompanied by pictures. Their task was to decide how well each sentence described the situation in the corresponding picture. The sentences contained plural nouns and they either matched or mismatched the pictures depending on whether the critical noun was interpreted inclusively or exclusively. An example of a stimulus sentence is given below.

**Table:** 

(8)	Each bed with a headboard is decorated with pillows.

The sentence was accompanied by a picture showing three beds with a headboard, two of which had multiple pillows and one had a single pillow, so the sentence was true of the picture only under an inclusive reading of the noun *pillows*. Plural nouns were placed either in the restrictor (a downward monotone position) or the nuclear scope (an upward monotone position) of the quantifier *each*.^5^ According to the monotonicity account of plural interpretation, an exclusive reading should be more likely for plural nouns in the nuclear scope position. This prediction was borne out. Single items were rejected more often as potential referents for a plural in the nuclear scope condition in comparison to the restrictor condition. However, the overall exclusive plural response rates in the nuclear scope, although higher than in the restrictor, were relatively low (34%, 26% and 38% of responses in first three experiments). When the authors used unquantified versions of sentences, the rate of responses consistent with the exclusive interpretation increased to 73%. Given that both environments (the unquantified sentence and the nuclear scope of the quantifier *each*) were upward monotone, Anand and colleagues concluded that monotonicity plays an important role, but is likely not the only factor relevant for the interpretation of plural nouns.

Spector (2007) offers a scalar-implicature account of plural meaning according to which the “basic” interpretation of plural nouns is inclusive (“one or more”). Singular nouns, on the other hand, restrict their reference to single atomic individuals.^6^ Thus, singular nouns, as more specialized forms, should be selected when the speaker wants to talk about an atomic object. If a plural noun is used instead, comprehenders can assume that the intended reference does not include atoms and thus the exclusive (“two or more”) reading of the plural emerges. This analysis accounts for the inclusive reading under negation and in other downward monotone contexts because inferences are less likely to arise in such environments (Frazier 2008). The exclusive interpretation of plurals appearing there is weakened or cancelled. The scalar-implicature hypothesis was tested experimentally by Tieu et al. (2014) using a truth-value judgment task. They asked children and adult participants to evaluate the truth of statements referring to short stories told by the experimenter. Critical words in the statements were singular and plural nouns and the statements were either affirmative (upward monotone) or negative (downward monotone). The results indicated that both age groups computed more exclusive plural interpretations in affirmative than in negative conditions, which replicated the results of Anand et al. (2011). Crucially, children were significantly less likely to assign exclusive readings to plurals in affirmative (upward monotone) sentences than adults. Because children have been independently demonstrated to be less capable of properly using scalar implicatures (Papafragou and Musolino 2003), this outcome was taken to support the implicature model of plural interpretation.

There is some evidence suggesting that the two readings may get activated simultaneously during comprehension, competing for selection. Patson (2016) conducted a picture-matching experiment investigating the mental representations of plural noun meanings. Participants had to decide whether the objects represented on pictures were mentioned in preceding written sentences. All experimental sentences contained plural nouns whose referents were characterized by the sentential context as a set of objects that were either spatially gathered (e.g., *rake up the leaves*) or spatially distributed (e.g., *scatter the leaves*). The images similarly depicted spatially grouped or scattered sets of objects but some also represented single objects. When the arrangement of the set on the picture did not match the meaning of the sentence, the response times were longer in comparison to matching trials. This suggests that comprehenders constructed a relatively detailed representation of the meaning of plural expressions. Interestingly, pictures with a single object did not differ significantly from pictures with a matching set. This was taken by the author as evidence that the “one or more” reading was activated along with the “two or more” reading and led to the facilitation of responses to pictures of single objects (e.g., a picture of a single leaf) following plural expressions (e.g., *the leaves*). However, this effect may also result from the simple fact that a plurality is a collection of individuals, so a picture of a single leaf may be seen as a subset or fragment of a stack of leaves.

### Negation and Parser Incrementality

An important area of investigation in psycholinguistics is parser incrementality. The central question here is whether during language comprehension a semantic interpretation is being assigned online as new words become available or whether the semantic analysis is attempted only after the entire sentence has fully unfolded and its syntactic structure has been computed. Evidence from psycholinguistic studies seems to support the first possibility. In an eye-tracking experiment conducted by Tanenhaus et al. (1995), participants directed their gaze at visually displayed objects immediately after hearing the words used to describe those objects, instead of waiting for the clause to unfold completely. This automatic interpretation of linguistic expressions was affected very early by context, including extra-linguistic information. Kamide et al. (2003) demonstrated that comprehenders compose the meaning of an expression as it is unfolding. This incrementally built interpretation of a sentence fragment can be used to predict what might come next, like anticipating the properties of the object from the semantics of the verb plus the subject.

However, apparently not every kind of information is equally rapidly integrated with the incrementally built interpretation. In an ERP study, Fischler et al. (1983) found an increased N400 (an ERP component associated with semantic processing) for the last word of false affirmative sentences with respect to true affirmative sentences.

**Table:** 

(9)	A robin is a bird.	[true]
(10)	A robin is a tree.	[false][increased N400]

In contrast, false negative sentences did not elicit an increased N400 in comparison to true negative sentences. In fact, the N400 effect was reversed for negative sentences, with the logically true sentences showing a bigger N400 amplitude than false sentences.

**Table:** 

(11)	A robin is not a bird.	[false]
(12)	A robin is not a tree.	[true][increased N400]

According to the authors, this result suggests that the interpretation of a negative expression proceeds in two steps: the affirmative version of a negated sentence is evaluated first, before the whole proposition is negated. At the first stage of comprehension sentences (9) and (11) are equivalent.^7^

This hypothesis received support from the outcome of a more recent ERP study by Lüdtke et al. (2008). The participants read affirmative and negative sentences, like the example below.

**Table:** 

(13)	In the front of the tower there is a ghost/no ghost.

Each sentence was followed after a delay by an image depicting either the object named in the sentence or an unrelated object. The sentence-image delay was either short (250 ms) or long (1500 ms). There was a priming effect (reduced N400) for pictures with related objects after both affirmative and negative sentences. That result was consistent with the possibility that comprehenders build an early representation of the meaning of a negative sentence ignoring the impact of negation. The phrase *a ghost* and the phrase *no ghost* similarly primed the picture of a ghost. The priming effect was observed regardless of the sentence-picture delay. However, manipulating the delay did influence the effect of negation in a different way. With a shorter delay, a difference in the EEG recording between affirmative and negative sentences was detected in a relatively late time window (starting around 550 ms after picture onset). With a longer delay, an affirmative/negative difference appeared during a considerably earlier time window (starting around 250 ms after picture onset). This was taken as evidence that negation needed some time to be fully integrated into the sentence interpretation. Only after a sufficiently long sentence-picture delay was negation information available early on for the verification task decision (although still unable to cancel the lexical priming effect). A related observation concerning the impact of negation on ERP components can be found in Kutas and Federmeier (2011): “[In] some cases (e.g., negation in the absence of pragmatic licensing), information that ultimately impacts plausibility judgments is not active in time to modulate N400 activity” (p. 633). Kaup et al. (2006) presented additional experimental evidence that computing the full representation of a situation described by a negative sentence requires extra time. This idea is sometimes referred to as the two-step simulation hypothesis of negation processing. A further discussion can be found in Spychalska (2011).

If the two-step simulation hypothesis is correct and delaying the semantic contribution of negation is common during language comprehension, the influence of a negative operator on the interpretation of individual words in the sentence should not be immediate. Consequently, the conversion from the exclusive to inclusive plural reading should take place at a later stage, perhaps during sentence-level information integration. The present study explored this possibility using a design based on the numerical Stroop interference for grammatical number.

## Numerical Stroop Interference

As discussed above, a plural noun occurring in downward monotone contexts (e.g., under negation or certain types of quantification) receives typically an inclusive, number-neutral reading. This is in contrast to the exclusive reading that plurals receive in more frequent upward-monotone contexts (e.g., unquantified declarative affirmative sentences). Is the inclusive interpretation assigned to the word immediately when it is encountered or is it a property emerging at the level of the compositional interpretation of the entire sentence? An answer to this question requires a research method sensitive to number semantics and capable of providing some information about the early, possibly automatic process of number value extraction from a noun during language comprehension. One technique satisfying these requirements is a number-related variant of the Stroop effect.

Broadly understood, a Stroop interference is a difficulty with response to conflicting information coming from different sources. The classic Stroop effect occurs in experiments with participants naming the visual color of color words like *red*. When the color of the font does not match the color name (e.g., the word *red* written in green font), participants’ responses are longer than in a congruent condition (Jaensch 1929; Jensen and Rohwer 1966; MacLeod 1991; Stroop 1935). In a numerical variant of the effect, counting instances of number symbols (digits or numerals) presented visually on a card or a screen takes more time when their visual numerosity is incongruent with their numerical value (e.g., symbol *2* repeated four times: *2 2 2 2*) than in a congruent condition (Flowers et al. 1979; Naparstek and Henik 2010; Pavese and Umiltà 1998; Windes 1968). A similar interference effect has been used by Berent et al. (2005) to investigate the processing of grammatical number. In a series of experiments, the authors presented singular and plural words to native Hebrew speakers. Sometimes a single copy would be visible on the screen (a visually single condition) and sometimes two copies would appear simultaneously (a visually double condition). Sequences of repeated letters formed the baseline condition. The participants were asked to decide for every trial whether they see one or more than one word and indicate their decision by pressing a key. Grammatical number interfered with the ability to count visually presented words. When the grammatical number of the word was incongruent with the visual numerosity, the participants’ responses were significantly slower than for the congruent trials, which was a form of a numerical Stroop interference. The authors interpreted this outcome as suggesting that number value is extracted automatically from lexical forms. Interestingly, this effect was found for grammatically plural words only. When a word with a plural marker was presented as visually single (e.g., *dogs*), the responses were longer than when it was presented as visually double (e.g., *dogs dogs*). Singulars did not differ significantly from the baseline. This outcome of the experiment indicated that participants treated plural nouns in isolation from a sentential context as having an exclusive (“two or more”) interpretation. Singulars in isolation seemed semantically unspecified for number.

Patson and Warren (2010) modified the Stroop technique described by Berent et al. (2005) and used it to investigate a specific phenomenon in grammatical number comprehension. First, they demonstrated that the interference between linguistic number and visual numerosity can be observed for words presented in sentential contexts. Participants read sentences displayed in a self-paced reading format in one- or two-word chunks and decided how many words were present in the final chunk. In critical trials, the final fragment was always a single word (a singular or plural noun). Responses to plural nouns were significantly longer than to singular nouns. This replicated the effect observed previously by Berent et al. (2005) for words in isolation. In another experiment reported in this study, the same technique was used with sentences containing singular nouns in the scope of a distributive or collective operator.

**Table:** 

(14)	**Each of** the men carried a box.	[distributive]
(15)	**Together** the men carried a box.	[collective]

This was done to test the possibility that singular nominals in distributive contexts could be treated as conceptually plural. The results suggested that this was indeed the case. When a singular noun was in a distributive predicate, participants needed more time to decide that they saw one word on the screen in comparison to a singular noun in a collective predicate. The authors concluded that the numerical interpretation of a singular noun can be affected by its sentential context (in particular by quantifiers) and that this happens during a relatively early comprehension stage.

## Present Study: Research Problem, Hypotheses and Predictions

Is the early automatic interpretation of a plural noun in the scope of sentential negation inclusive or exclusive? The present experiment explored that issue using a design based on the numerical Stroop effect by placing plural nouns in affirmative sentences and their negated versions.

### **Hypothesis 1**

The default interpretation of plural nouns is exclusive and it is also their initial reading in upward monotone environments (Anand et al. 2011; Tieu et al. 2014). Under this reading, plural nouns refer to a group of two or more entities. This conceptual plurality should create an interference with the visual number for plurals presented on the screen as one item and a facilitation for plurals displayed as two copies visible simultaneously. Plural nouns occurring in affirmative sentences were, therefore, predicted to be easier to count in the visually double condition than in the visually single condition. The effect should resemble the interference observed by Berent et al. (2005) for plural words presented in isolation with no sentential context.

### **Hypothesis 2**

The initial reading of plural nouns in negative sentences should not differ significantly from affirmative sentences. Although plural nouns are typically interpreted inclusively in downward monotone environments (like the scope of sentential negation), the results of previous studies on negation (Fischler et al. 1983; Lüdtke et al. 2008) suggest that the compositional integration of negation with the rest of the sentence may be delayed. According to the two-step simulation hypothesis of negation processing, the affirmative version of a sentence is evaluated first before the negated version is computed (Kaup et al. 2006; Spychalska 2011). Plural nouns occurring in negative sentences were, therefore, predicted to be easier to count in the visually double condition than in the visually single condition.

## Experiment

The present experiment tested the hypotheses described above. During the experiment, sentences ending with a noun were displayed in one- or two-word chunks on the screen. The final noun was displayed either once or repeated twice. Participants were instructed to count the number of noun instances in the final fragment. Reaction times and accuracy were measured.

### Materials

The critical items consisted of 60 nouns:

- 30 singular nouns (e.g., *królik* “rabbit”)
- 30 plural forms created from the singulars (e.g., *króliki* “rabbits”)

The words were embedded in 60 affirmative sentences, illustrated with examples in (16), and in their negative versions, illustrated in (17).

**Table:** 

(16)	a	Adam	widział	małego	**królika.**
		Adam	see.3sg.pst.ipfv	small.sg.acc	rabbit.sg.acc
		“Adam saw a small rabbit.”			
	b	Adam	widział	małe	**króliki.**
		Adam	see.3sg.pst.ipfv	small.pl.acc	rabbit.pl.acc
		“Adam saw small rabbits.”

**Table:** 

(17)	a	Adam	nie	widział	żadnego	**królika.**
		Adam	neg	see.3sg.pst.ipfv	any.sg.gen	rabbit.sg.gen^8^
		“Adam did not see any rabbit.”				
	b	Adam	nie	widział	żadnych	**królików.**
		Adam	neg	see.3sg.pst.ipfv	any.pl.gen	rabbit.pl.gen
		“Adam did not see any rabbits.”				

The experimental noun was always sentence-final and it was preceded by an adjective (in affirmative sentences) or by the word *żaden* (in negative sentences).^9^ See “Appendix” for the list of critical sentences.

Additionally, 40 filler sentences were created (20 affirmative and 20 negative), all ending with an adverb (e.g., *szybko* “fast”).

**Table:** 

(18)	Lidka	jechała	bardzo	**szybko.**
	Lidka	drive.3sg.pst.ipfv	very	fast

“Lidka drove very fast.”

**Table:** 

(19)	Lidka	nie	jechała	wcale	**szybko.**
	Lidka	neg	drive.3sg.pst.ipfv	at.all	fast
	“Lidka did not drive fast at all.”				

### Procedure

**Fig. 1:**
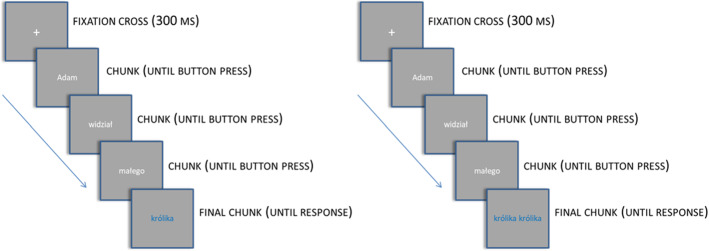
The structure of a trial in the visually single and visually double condition

The procedure was based on the experiment presented in Patson and Warren (2010), which itself was a modified version of the technique used in Berent et al. (2005). Each sentence was introduced by a fixation cross which remained on the screen for 300 ms. Sentences were presented in one- or two-word chunks displayed at the center of the screen. The participants moved to the next chunk by pressing the space bar. The last chunk was always displayed in blue font and it was either a single word (e.g., *królika* “rabbit”) or the same word repeated twice (e.g., *królika królika* “rabbit rabbit”) (Fig. 1). The participants were instructed to decide how many blue font words they see on the screen at the end of each sentence by pressing the left arrow key (one word) or the right arrow key (two words).^10^ On 56 out of 320 trials (balanced across conditions) the sentence was followed by a comprehension question presented in green font with two possible answers displayed below the question on the left and right side of the screen. The questions concerned the verb, object, adjective or the meaning of the whole sentence (see Table 4) The participants indicated their choice by pressing the left or right arrow key.

The experiment proper was preceded by instructions and a training session consisting of 14 sentences with four comprehension questions. A feedback message was displayed after every answer informing whether the answer was correct or incorrect. In the experiment proper, a feedback message appeared only after an incorrect response. The trial session ended with a message informing participants about the number of correct and incorrect responses. No training item appeared later in the experiment.

Halfway through the experiment there was a message informing participants about a break. The participant could proceed when ready by pressing the space bar. A single experimental session lasted around 20 min.

The experiment was designed and presented using the PsychoPy software, version 1.83.03 (Peirce 2007, 2009).

### Participants

Thirty-one students of the Institute for English Studies of the University of Wrocław (8 men, 23 women) took part in the experiment. Participants were all native speakers of Polish. The average age was 19.9 (*SD *= 1.27).

### Results: Reaction Times

For the response times (RT) analysis, the data were cleaned first by removing all incorrect responses and then by eliminating all trials with response times 2 standard deviations above or below the mean for each participant in each condition. This resulted in removing 5.1% of correct trials, distributed roughly equally across participants and conditions. Statistical analysis was performed with the SPSS software, version 22.

A **2 × 2×2 ANOVA** was conducted with RT as the dependent variable and the following independent factors:

- Polarity (affirmative, negative)
- Grammatical Number (singular, plural)
- Visual Number (visual 1, visual 2)

There was no main effect of Polarity (*F*_1_(1,30) = 0.62 *p *= .436; *F*_2_(1,116) = 0.65 *p *= .421) or Grammatical Number (*F*_1_(1,30) = 0.72 *p *= .404; *F*_2_(1,116) = 1.25 *p *= .265). The main effect of Visual Number was not significant by subjects (*F*_1_(1,30) = 1.70 *p *= .203) but it was significant by items (*F*_2_(1,116) = 8.50 *p *< .01 η_p_^2^ = .07). Items presented on the screen as visually single were on average responded to more slowly than items presented as visually double, as shown in Table 1.

**Table 1:** Mean reaction times and accuracy in the counting task for nouns presented in the visually single and visually double condition (standard errors in parentheses)

Visual number		RT (ms)	Accuracy (% correct)
Visual 1	*pojazd* “vehicle”	647 (24)	98.5
Visual 2	*pojazd pojazd*	637 (25)	98.6

The Polarity × Grammatical Number interaction was not significant (*F*_1_(1,30) = 0.58 *p *= .452; *F*_2_(1,116) = 0.70 *p *= .405) and neither was the interaction of Polarity × Visual Number (*F*_1_(1,30) = 0.12 *p *= .728; *F*_2_(1,116) = 0.03 *p *= .868).

The interaction of Grammatical Number × Visual Number was significant both by subjects (*F*_1_(1,30) = 8.34 *p *< .01 η_p_^2^ = .22) and by items (*F*_2_(1,116) = 14.69 *p *< .001 η_p_^2^ = .11). Responses to singular nouns were on average faster in the visually single condition than in the visually double condition. The pattern was reversed for plural nouns. This congruency effect was larger for plural nouns (see Table 2).

**Table 2:** Mean reaction times and accuracy in the counting task for singular and plural nouns in the visually single and visually double condition (standard errors in parentheses)

Grammatical number		Visual number					
		Visual 1		Visual 2		Congruency	
						(Visual 1–Visual 2)	
		RT (ms)	Accuracy (% correct)	RT (ms)	Accuracy (% correct)	RT (ms)	Accuracy (% correct)
Singular	*pojazd* “vehicle”	637 (23)	98.9	641 (25)	98.6	− 4	0.3
Plural	*pojazdy* “vehicle”	656 (26)	98.1	632 (24)	98.7	24	− 0.6

**Fig. 2:**
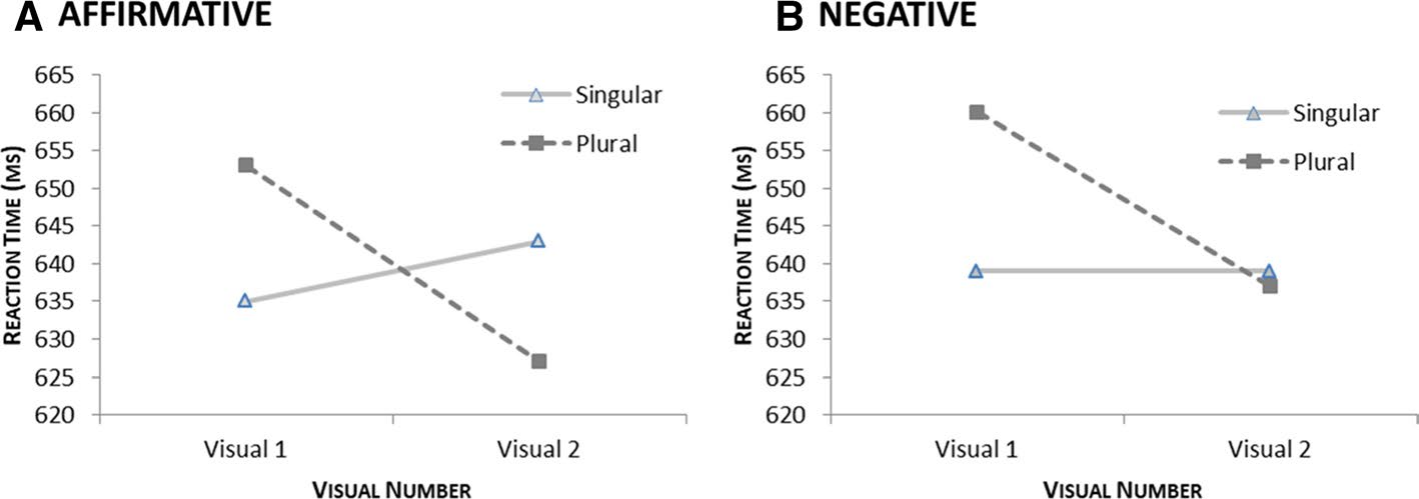
Congruency effect (Stroop-like interference) of grammatical number and visual numerosity in affirmative (left diagram) and negative (right diagram) sentences

Crucially, the three-way interaction of Polarity × Grammatical Number × Visual Number was not significant (*F*_1_(1,30) = 0.23 *p *= .637; *F*_2_(1,116) = 1.34 *p *= .250), indicating that the manipulation of the visual numerosity had roughly the same effect on nouns in affirmative and negative sentences. This was confirmed by an inspection of the data (see Table 3 and the graphs in Fig. 2). If anything, counting singular nouns was more sensitive to monotonicity than counting plural nouns as the congruency effect was noticeably bigger in affirmative than in negative sentences for singulars. However, given that the overall interaction was not significant and that our predictions concerned only plural nouns, no explanation for this trend is offered here.

**Table 3:** Mean reaction times and accuracy in the counting task for singular and plural nouns in affirmative and negative sentences in the visually single and visually double condition (standard errors in parentheses)

Polarity	Gram. number	Visual number					
		Visual 1		Visual 2		Congruency	
						(Visual 1–Visual 2)	
		RT (ms)	Accuracy (% correct)	RT (ms)	Accuracy (% correct)	RT (ms)	Accuracy (% correct)
Affirmative	Singular	635 (22)	98.9	643 (25)	98.2	− 8	0.7
	Plural	653 (28)	97.7	627 (25)	98.5	26	− 0.8
Negative	Singular	639 (25)	98.9	639 (26)	99	0	− 0.1
	Plural	660 (25)	98.4	637 (25)	98.8	23	− 0.4

### Results: Comprehension Questions

In our experiment the visually double trials contained the same word repeated twice, which was not the case for the experiment described in Patson and Warren (2010) (see footnote 12). It could be argued that the introduction of the word-doubling made the materials too artificial and resulted in a task strategy whereby the sentences were simply not interpreted compositionally. In this case, the absence of a polarity effect might not reflect the delayed interpretation of negation, but the shallow (non-compositional) processing of most sentences in this study. In particular, participants may have ignored the marker of negation, jumping ahead to the last chunk of the sentence to count the words. To investigate this possibility, the accuracy in comprehension questions was checked. Results are given in Table 4.

**Table 4:** Average accuracy for different types of comprehension questions (standard deviations in parentheses)

Question type		Accuracy (% correct)
Adjective question	*Jakich bandytów ścigał policjant?* “What kind of bandits did the policeman chase?”	99.2% (4.5)
Adverb question	*Jak pracował górnik?* “How did the miner work?”	99.5% (2.6)
Object question	*Co widział Adam?* “What did Adam see?”	99.1% (3.1)
Sentence question	*Czy malarz czyścił pędzel?* “Did the painter clean the brush?”	90.1% (10.4)
Verb question	*Co robiła Magda?* “What did Magda do?”	97.7% (4.9)

Overall, answers indicated that participants paid attention to the contents of the sentences. While it is true that answers to the questions concerning the whole sentence were less accurate than to other question types, the accuracy for this type was still high (over 90%). This suggests that participants were attentive to the sentence polarity.

## Discussion

The experiment described in the present paper was designed and conducted to investigate the immediate interpretation of plural nouns in the scope of sentential negation. Plurals in negative sentences, as in other downward monotone environments, are typically given an inclusive reading (“one or more”), which contrasts with their more frequent exclusive reading (“two or more”). The research question investigated in the present study was whether the inclusive reading is assigned to a plural noun in the scope of sentential negation immediately when it is encountered or whether this reading originates from a delayed interpretation of the entire sentence. To address this question we used an experimental method based on a variant of the Stroop interference effect. The technique was first applied to study grammatical number processing for words in isolation by Berent et al. (2005) and later extended to words presented in context by Patson and Warren (2010).

Our first hypothesis was that the basic reading of a plural word should be exclusive. This reading is prevalent in upward monotone environments (e.g., unquantified affirmative declarative constructions). The second hypothesis was that in negative constructions, the change from the exclusive to inclusive reading should take place after some delay, since negation seems to require extra time to be fully semantically processed, as indicated by previous research (Fischler et al. 1983; Kaup et al. 2006; Lüdtke et al. 2008). We predicted, therefore, that plural nouns in both the affirmative and negative conditions should give rise to a comparable Stroop effect. This prediction was borne out. Participants were slower to count plural nouns when they were presented as a single word than when they were repeated twice on the screen (consistent with the exclusive reading). This was true for both affirmative and negative sentences and there were no statistically significant differences between the two conditions.

The results of the present experiment are in line with the two-step simulation hypothesis of negation processing (Kaup et al. 2006; Lüdtke et al. 2008; Spychalska 2011). According to the hypothesis, the semantic contribution of negation is delayed so that a language comprehender evaluates first the affirmative variant of a negative sentence before negating the meaning at a later stage. In effect, when a plural noun is encountered in a negative sentence (e.g., *I have not seen rabbits*) it is first treated as if occurring in an affirmative sentence (e.g., *I have seen rabbits*), receiving an exclusive reading. The results provide support for the idea that language comprehension mechanisms, although geared towards a rapid incremental compositionality (Kamide et al. 2003; Tanenhaus et al. 1995), can delay the semantic contribution of some elements until a later processing phase.

Although we focused specifically on the processing of plural nouns, the results for singular nouns in the present experiment are also worth discussing. The pattern observed in responses for singular nouns was directly opposite to the pattern observed for plurals. Participants needed on average more time to count singular nouns when they were repeated twice on the screen than when only one copy was displayed. This is an expected Stroop interference, assuming that singular nouns activate a concept of singularity, which makes the counting of multiple visually presented items more difficult. In Berent et al. (2005), singular nouns did not give rise to any interference effect, which can be interpreted as a lack of number specificity for singular forms. The idea that singular nouns are number neutral has been proposed in the theoretical literature (Farkas and de Swart 2010) and is consistent with the singular/plural asymmetry found in experimental studies investigating agreement attraction (Bock and Miller 1991; Pearlmutter et al. 1999). The present results seem at odds with those previous findings. A possible explanation might lie in the morphological form of the nouns used as stimuli. There is some evidence that the strength of the activation of a number concept for singular nouns may be related to markedness, more specifically to the presence or absence of an overt number morpheme (Gulgowski and Błaszczak 2018). In the present experiment, the majority of singular nouns (and all plural nouns) were marked with an overt case/number ending. The type of morphological marking of number is seldom explicitly discussed or controlled, but the apparent lack of specific number meaning for singular nouns in the past studies may have resulted from using mostly unmarked singular forms (which is especially likely for studies with English stimuli). It is worth noticing that the congruity effect between grammatical number and visual numerosity was considerably larger for plural than for singular nouns (see Table 2), suggesting a stronger connection with a specific number meaning for the former.

## Appendix

**Table:**

#	Affirmative sentences
1	*Adam widział małego królika/małe króliki*
2	*Magda poganiała swojego kolegę/swoich kolegów*
3	*Renata słyszała znanego muzyka/znanych muzyków*
4	*Artur karmił swojego chomika/swoje chomiki*
5	*Bożena polecała wybitnego pisarza/wybitnych pisarzy*
6	*Marek oczekiwał swojego sąsiada/swoich sąsiadów*
7	*Janek chwalił bystrego studenta/bystrych studentów*
8	*Justyna witała zagranicznego artystę/zagranicznych artystów*
9	*Policjant ścigał groźnego bandytę/groźnych bandytów*
10	*Piotrek odwiedzał swojego krewnego/swoich krewnych*
11	*Lucyna odganiała natrętnego komara/natrętne komary*
12	*Chłopiec gonił szarego szczura/szare szczury*
13	*Gospodarz strzygł białego barana/białe barany*
14	*Lekarz badał chorego pacjenta/chorych pacjentów*
15	*Kłusownik tropił rannego tygrysa/ranne tygrysy*
16	*Kelner wycierał srebrny widelec/srebrne widelce*
17	*Janek wybierał tani skuter/tanie skutery*
18	*Mechanik testował nowy silnik/nowe silniki*
19	*Krawiec szył modny sweter/modne swetry*
20	*Agata niosła ciężki plecak/ciężkie plecaki*
21	*Malarz czyścił swój pędzel/swoje pędzle*
22	*Antek czytał ciekawy magazyn/ciekawe magazyny*
23	*Uczeń strugał swój ołówek/swoje ołówki*
24	*Olga kupowała drogiego laptopa/drogie laptopy*
25	*Praczka prała brudny szalik/brudne szaliki*
26	*Maria odnawiała stary kredens/stare kredensy*
27	*Rolnik oglądał zepsuty traktor/zepsute traktory*
28	*Łukasz szorował tłusty garnek/tłuste garnki*
29	*Paulina myła swój talerz/swoje talerze*
30	*Kierowca tankował swój pojazd/swoje pojazdy*

**Table:**

#	Negative sentences
1	*Adam nie widział żadnego królika/żadnych królików*
2	*Magda nie poganiała żadnego kolegi/żadnych kolegów*
3	*Renata nie słyszała żadnego muzyka/żadnych muzyków*
4	*Artur nie karmił żadnego chomika/żadnych chomików*
5	*Bożena nie polecała żadnego pisarza/żadnych pisarzy*
6	*Marek nie oczekiwał żadnego sąsiada/żadnych sąsiadów*
7	*Janek nie chwalił żadnego studenta/żadnych studentów*
8	*Justyna nie witała żadnego artysty/żadnych artystów*
9	*Policjant nie ścigał żadnego bandyty/żadnych bandytów*
10	*Piotrek nie odwiedzał żadnego krewnego/żadnych krewnych*
11	*Lucyna nie odganiała żadnego komara/żadnych komarów*
12	*Chłopiec nie gonił żadnego szczura/żadnych szczurów*
13	*Gospodarz nie strzygł żadnego barana/żadnych baranów*
14	*Lekarz nie badał żadnego pacjenta/żadnych pacjentów*
15	*Kłusownik nie tropił żadnego tygrysa/żadnych tygrysów*
16	*Kelner nie wycierał żadnego widelca/żadnych widelców*
17	*Janek nie wybierał żadnego skutera/żadnych skuterów*
18	*Mechanik nie testował żadnego silnika/żadnych silników*
19	*Krawiec nie szył żadnego swetra/żadnych swetrów*
20	*Agata nie niosła żadnego plecaka/żadnych plecaków*
21	*Malarz nie czyścił żadnego pędzla/żadnych pędzli*
22	*Antek nie czytał żadnego magazynu/żadnych magazynów*
23	*Uczeń nie strugał żadnego ołówka/żadnych ołówków*
24	*Olga nie kupowała żadnego laptopa/żadnych laptopów*
25	*Praczka nie prała żadnego szalika/żadnych szalików*
26	*Maria nie odnawiała żadnego kredensu/żadnych kredensów*
27	*Rolnik nie oglądał żadnego traktora/żadnych traktorów*
28	*Łukasz nie szorował żadnego garnka/żadnych garnków*
29	*Paulina nie myła żadnego talerza/żadnych talerzy*
30	*Kierowca nie tankował żadnego pojazdu/żadnych pojazdów*

## Footnotes

1 But see, for instance, Booij (1993) for a discussion of plural nouns participating in word-formation processes, which is typical for derived rather than inflected forms.

2 Some languages seem to possess the category of verbal number (also known as pluractionality). It can be characterized as reference to multiple events through the use special verb forms. The distinction between verbal number and aspect is not always clear though. For a discussion of pluractionality, see Durie (1986), Corbett (2000) or Hofherr (2010).

3 Number forms can also acquire special secondary uses, expressing concepts like emphasis and abundance or referring metonymically to the inhabitants of a place (Corbett 2000).

4 For more information see, among others, Nouwen (2010), Penka and Zeijlstra (2010), Spector (2007) or Tunstall (1998). See also discussions regarding the controversial monotonicity status of interrogative sentences (Giannakidou 1998; Gutiérrez-Rexach 1997; Progovac 1993; van der Wouden 1997) and conditionals (Gajewski 2011; Heim 1984; von Fintel 1999).

5 Quantification in natural languages refers to the means for expressing relations between sets. Quantifiers are commonly described in terms of a tripartite structure: the operator, restrictor and nuclear scope (Partee 1991). The restrictor introduces the primary set and the nuclear scope provides another set that stands in some relation to the restrictor. For the quantifier *each* the restrictor is downward monotone and the nuclear scope is upward monotone.

**Table:**

	operator	restr.	n. scope		operator	restr.	n. scope
(i)	*Each*	***fruit***	*is sweet*	→	*Each*	***apple***	*is sweet*
	*Each*	***apple***	*is sweet*	↛	*Each*	***fruit***	*is sweet*
(ii)	*Each*	*basket*	***contains a fruit***	↛	*Each*	*basket*	***contains an apple***
	*Each*	*basket*	***contains an apple***	→	*Each*	*basket*	***contains a fruit***

6 Spector (2007) defines atomic individuals as “individuals whose only parts are themselves” (p. 10). Atomic individuals contrast with mereological sums. Individual z is a sum of individuals x and y if “x is a part of z, y is a part of z, and z is a part of any z’ that also contains x and y as parts” (p. 10).

7 It is possible that the N400 component is sensitive to lexical relations between words and is simply not affected by compositional processes. A comparable N400 for both *A robin is a bird* and *A robin is not a bird* may result from a strong relation between the words *robin* and *bird*. This is consistent with the results from some of the more recent studies. For example, Hoeks et al. (2004) reported no N400 amplitude increase for syntactically well-formed sentences with semantically anomalous order of arguments (e.g., *The javelin has thrown the athletes*) in comparison to sentences with no semantic anomaly. Instead, the anomalous argument order gave rise to a P600 effect. However, the picture may be more complex. In a study by Vissers et al. (2013), an increased N400 amplitude appeared for argument-anomalous sentences (e.g., *The fox that hunted the poachers stalked through the woods*) as a function of mood. This suggests that structural relations may influence semantic processing in the N400 time window under certain conditions. For more discussion, see Brouwer et al. (2012) or Chow and Phillips (2013). See also the delay experiment described in this section.

8 The direct object of a negated transitive verb in Polish is obligatorily marked for Genitive, a phenomenon known as *Genitive of Negation* (Błaszczak 2001a, b, Blaszczak 2007; Przepiórkowski 1997; Witkoś 1998).

9 The word *żaden* is similar to English *any* in that it is licensed by sentential negation (see Błaszczak 2001b, for a detailed discussion). It is inflected like an adjective and it was chosen as an additional marker of negation to strengthen the possible effect.

10 In Patson and Warren (2010) the last word was never doubled, instead the final chunk contained either the last word presented as a single token (visually single) or the last two words of the sentence (visually double). However, with this setup the participant is not guaranteed to pay attention to the noun itself, possibly focusing instead on the penultimate word. The doubling in our study was introduced to make sure that the participant is counting nouns. By having two plural nouns visible on the screen simultaneously we hoped to increase the salience of grammatical number.
